# Gender Diversity in STEM Disciplines: A Multiple Factor Problem

**DOI:** 10.3390/e21010030

**Published:** 2019-01-04

**Authors:** Carmen Botella, Silvia Rueda, Emilia López-Iñesta, Paula Marzal

**Affiliations:** 1 School of Engineering (ETSE-UV), Universitat de València, Av. De l’Universitat s/n, 46100 Valencia, Spain; 2 Department of Didactics of Mathematics (Faculty of Teaching), Universitat de València, Av. Tarongers, 4, 46022 Valencia, Spain

**Keywords:** women in STEM, gendered innovation, gender diversity

## Abstract

Lack of diversity, and specifically, gender diversity, is one of the key problems that both technological companies and academia are facing these days. Moreover, recent studies show that the number of female students enrolled in science, technology, engineering and mathematics (STEM) related disciplines have been decreasing in the last twenty years, while the number of women resigning from technological job positions remains unacceptably high. As members of a higher education institution, we foresee that working towards increasing and retaining the number of female students enrolled in STEM disciplines can help to alleviate part of the challenges faced by women in STEM fields. In this paper, we first review the main barriers and challenges that women encounter in their professional STEM careers through different age stages. Next, we focus on the special case of the information theory field, discussing the potential of gendered innovation, and whether it can be applied in the Information Theory case. The working program developed by the School of Engineering at the University of Valencia (ETSE-UV), Spain, which aims at decreasing the gender diversity gap, is then presented and recommendations for practice are given. This program started in 2011 and it encompasses Bachelor, Master and PhD levels. Four main actions are implemented: Providing institutional encouragement and support, increasing the professional support network, promoting and supporting the leadership, and increasing the visibility of female role models. To assess the impact of these actions, a chi-square test of independence is included to evaluate whether there is a significant effect on the percentage of enrolled female students. The percentage of graduated female students in the information and Communications Technology Field is also positioned with respect to other universities and the Spanish reference value. This analysis establishes that, in part, this program has helped to achieve higher female graduation rates, especially among Bachelor students, as well as increasing the number of top-decision positions held by faculty women.

## 1. Introduction

The information and communications technology (ICT) sector has been growing at a quick pace for the last twenty years. This technological sector is highly dynamic and shows tremendous potential for innovation and for introducing changes impacting deeply on the society in the short-term. Traditionally, the ICT sector demands large numbers of graduates in science, technology, engineering, and mathematics (STEM) disciplines, and its employees are usually well paid. In this challenging environment, one may expect the sector to take advantage of as much brain power, creativity, and knowledge as possible. However, it fails to do so, since several studies indicate that it is a male-dominated sector in all stages of the professional development [1,2], with women facing a greater probability of working part-time, taking a break from their careers, or even resigning [2]. Women currently hold 21% of the high executive positions in the technology sector [1], while they represent only 13% of the highest positions if the engineering field is considered [3]. Of all ICT patents, 88% have been registered by all-male teams, and the gender pay gap is a reality, with women being paid from 18% to 22% less than men [4]. 

This situation is also reflected in academia and scientific research and development institutions, where women are under-represented in the top decision-making positions. In the case of the 28 countries comprising the European Union, for example, only 28% of scientific and administrative board members are women, and in 2014, women represented a mere 20% of the heads of higher education institutions [3]. Regarding the working conditions, 13% of women in research were part-time (2012) versus 8.5% of men. The gender pay gap is also visible here, with women’s earnings being 17.9% lower than those of men (2010) [3]. Women represent 33% of the researchers in the European Union, with a larger gender imbalance (less than 40% proportion) in the fields of engineering and technology and natural sciences [3].

Considering these numbers, it can be seen why increasing diversity in working teams in all professional stages has been a key issue for technological companies, as well as for academia. More precisely, gender diversity has been proved to increase the potential for innovation. In fact, defining teams with an equivalent composition in terms of women and men translates into increasing the creativity, the chances to experiment, the share of knowledge, and task fulfillment, with respect to building teams with other compositions [4]. Research on team composition states that the collective intelligence of the team is not a direct translation from the intelligence level of individual team members. Actually, a larger number of women in the teams can be related to higher levels of collective intelligence [4]. In parallel, research shows that an increased participation of women, not only in STEM fields, positively impacts the economy as a whole, as stated for example in Reference [5], which addresses the correlation of women empowerment and economic growth, or Reference [6], focusing on women’s roles and parliament presence for decreasing corruption.

Reality shows that the number of women enrolling in STEM related disciplines, and therefore, the chance to increase their numbers also in the job market and academia, is decreasing despite all efforts. For example, Reference [1] indicates that in 1991, 37% of computer science graduates were women, while this number went down to 26% in 2014 and to 18% in 2018. In the European Union [3], 25% of PhD graduates in engineering were women (2012), with 21% graduating in computing. In the case of Spain, for example, women represent 22% of the graduates in computing (2012), and 29% in engineering (2012) [3]. The Organization for Economic Co-operation and Development (OECD) states that less than a 20% of women register in STEM PhD programs, and only 18% start engineering studies, being 20% the average for computer science [7]. Similar numbers are given by the United Nations Educational, Scientific and Cultural Organization (UNESCO) in their report [8]. This document states that women stand for 35% of all students enrolled in STEM related disciplines in higher education. The lowest percentage (world average) is found in the areas of ICT (3%), engineering, manufacturing, and construction (8%), and natural science, mathematics and statistics (5%). What is more, women abandon in enormous numbers their higher education STEM studies, as well as during the transition to the job market and during their professional STEM career.

Interestingly, Reference [7] defines the point where the career path starts to diverge before 15 years old: On average, at that age, boys are more than twice as likely as girls to expect to work as scientists or engineers. Actually, less than 0.5% of girls would like to be working in the ICT sector, while this percentage increases to 5% of boys. With this perspective, some studies even suggest that the STEM gender gap starts from inborn differences between sexes regarding STEM aptitudes. However, this stereotype has been thoroughly revised in Reference [9], where the authors have analyzed possible gender differences in mathematical cognition from 500 children aged from six months to eight years. This analysis found that boys and girls do not show any difference in terms of quantitative or mathematical ability, thus they have the same aptitudes in terms of mathematical reasoning. Since the gender representation gap in STEM disciplines cannot be related to a matter of aptitudes, and it has been traced back to primary school, the vision is that we are dealing with a multiple factor problem, where several actors would need to impact at different age stages, as reflected in the following subsections. 

### 1.1. Barriers Encountered in the Professional Career

Women currently working in technological sectors find several barriers that prevent them from starting or progressing in their professional career. In a recent global survey carried out among women working in technology, the following aspects were highlighted as strong barriers [1]:

- 48% found there was a lack of mentors during their professional career
- 42% thought there was a lack of female role models
- 39% experienced gender bias in the workplace
- 36% pointed out that they had unequal growth opportunities compared to men
- 35% indicated that there was a gender pay gap for the same skills

Women consider that they lack the support of a strong professional network, and hence, they think that their networking opportunities are limited. When considering the question of why women are underrepresented in technology, the survey from Reference [1] found out that:

- 33% thought it was due to role models
- ICT was perceived as a male-dominated field (22%)
- There was a lack of balance between work and personal life (14%)
- There was not strong support and encouragement from educational institutions towards girls (14%)

From the above results, one can see that there are multiple factors related to the gender diversity problem, and even more, some of them appear at different life stages. These factors have been widely discussed in the scientific literature on gender equality, drawing on psychological evidence. For example, gender bias and the effects of sex stereotypes in the workplace are addressed by the authors of References [10,11,12,13], unequal growth opportunities in Reference [14], and the impact of social role models in Reference [15].

Traditionally, the work-life conflicts have been thought of as one of the main factors preventing women from progressing in the ICT sector [2,13]. Long working hours, career breaks related to motherhood, being available at the working place or even requirements related to travel, can be seen as sacrifices in the personal life with no professional gains. In this scenario, some companies and institutions are developing policies targeting flexible work schedules. However, it appears to be what has been called the flexibility stigma [1], meaning that flexible options should be a choice without negative consequences or penalties for women adhering to them. A recent study focusing on the Spanish job market [2], found out that, even though flexible job policies have been available in the ICT sector for many years, they do not seem to be the solution to increase the presence of women through all professional stages. In fact, the survey indicated that there were other variables related to stereotypes in the workplace about gender or age that were creating workplace dissatisfaction. The lack of inspiring role models, work environments not offering enough support, or the perception of the ICT working environment as male-dominated and aggressive (in terms of self-confidence), are seen as factors preventing women from entering the sector [2,4].

### 1.2. Challenges Faced at Different Age Stages

As pointed out before, the starting point of the gender gap in STEM disciplines can be located before the age of 15 years, and it is not due to differences in terms of mathematical reasoning. This means that there is a set of factors during primary school that needs to be analyzed and compensated for, with the collaboration of several institutions and actors. In our opinion, one step more would be required, achieving a change in our current society, leading to what can be regarded as a gender-sensitive society or culture. 

Gender roles, gender patterns, and stereotypes deeply installed in family and society about what careers are appropriate for both men and women have an impact on the future education of boys and girls, and their career choices [16]. One example to fight stereotypes can be seen in the case of toy retailers, where some research is done focusing on the catalogues [17]. In this case, simple recommendations such as including more mixed groups of boys and girls, more examples of children playing without stereotype, not to use ‘all girl’ or ‘all boy’ scenarios, and use colors without stereotype, can contribute to reducing the gender gap. At the institutional level, gender stereotyping at school has been addressed for example by the OECD through the Recommendation on Gender Equality in Education, Employment and Entrepreneurship, that was reflected in different programs depending on each country [7]. 

Focusing now on the professional age window, companies and institutions would benefit from adopting gender equality plans. These plans should be structured along the main axes of increasing the visibility of women in the technology field, promoting equal parenting, establishing flexible work arrangements, and reducing the gender wage gap. In the European Union case [3], 36% of the organizations performing research have adopted gender equality plans, including measures related to recruitment and promotion, flexible career trajectories, or support for leadership development. Moreover, gender equality is now part of the European research and innovation policy, with the aim of integrating the gender/sex (Gender refers to cultural and social determinants of feminine and masculine traits and behaviors. Sex is defined as chromosomal, hormonal, genetic and biological and a way to distinguish males from females [18]) dimension in the research content through what is known as gendered innovation, which is discussed in Section 2. 

Affirmative actions targeting gender equality can also infer a stigma of incompetence to women adhering to them, as reflected for example in References [19,20]. To avoid this, institutions adopting gender equality plans should not only focus on short-term criteria as increasing the number of women, for example, but they need to include the consideration of merits and qualifications and build a welcoming environment with support for women to achieve a long-term change in the diversity gap [19,20]. In the context of higher education institutions, actions related to gender equality need to integrate male students to avoid frustrated feelings in them, especially if they perceive that the female students are accessing exclusive resources [21].

The University of Valencia (UVEG), Spain, has adopted a gender equality plan with support for some of the actions that will be introduced in Section 3. Extracting the data from the last transparency report [22], Table 1 summarizes the percentage of women according to their academic position both at the University and at the School of Engineering (ETSE-UV). This table reflects that the presence of women decreases as we progress in the professional stages. Moreover, if we focus on the case of the ETSE-UV, where Engineering and Data Science studies are concentrated, the numbers are even more discouraging. Note that in Spain, the recruitment process for faculty positions is not currently implementing any mechanism to compensate gender-bias, while position promotion is solely related to the candidates’ curriculum and achievements.

In the following section, we will review the particular case of the Information Theory field, discussing the potential application of the gendered innovation initiative. In Section 3, we will introduce the working program towards decreasing the gender diversity gap carried out by the School of Engineering at the University of Valencia (ETSE-UV), Spain, targeting Bachelor, Master, and PhD levels of ICT-related disciplines. Section 4 presents then an exploratory analysis of the impact of these actions, evaluating a set of objective metrics with the aim of showcasing the efficacy of the program and supporting our recommendations for practice. Finally, conclusions are presented in Section 5.

## 2. The Information Theory Field: Impact of Gendered Innovations

The Information Theory field has been traditionally a male-predominant environment. In our opinion, one of the main reasons is the lack of visibility of women already working in the topic, discouraging other women to enter the field. This trend decreases the percentage of women, which also reduces their support network and can cause workplace dissatisfaction to arise in the end. 

A side factor contributing to this scenario may be the lack of practical applications of Information Theory from young girls’ perspective. Although results related to Information Theory have been fundamental for technology progress, their impact is not well-understood out of the field. Report [8] and Reference [23] reflect that interest, motivation, and altruistic values and attitudes are fundamental for increasing the number of students in STEM. An analysis carried out over 40 years focusing on occupational interests [24], showed that, independently of the time period and age window, men prefer working with things, and women prefer working with people. References [25,26,27] explore the goal congruity perspective, which frames the influence of social roles in motivational processes. Recently, in Reference [27], the STEM gender gap is addressed under this perspective, analyzing the impact of the perception of STEM fields as not achieving communal opportunities to work or help others. As the authors point out, understanding and specifically, transmitting the opportunities for achieving a communal goal offered by STEM disciplines can be seminal for decreasing the gender gap. Aligned with these pieces of evidence, we believe that highlighting the applicability is key to engage students in certain disciplines, as it is the case of Information Theory, where the final link to people needs to be emphasized outside the field. More precisely, two actions can boost the enrollment of women in the Information Theory field: Increasing the visibility of research in areas such as machine learning and data science and including the gender/sex analysis and gender diversity in the research dimension, which is known as gendered innovations [28].

In the actual massive data era, we are facing problems that require accessing loads of data, processing them to apply knowledge extraction techniques, and designing algorithms to analyze the data. On the other hand, it is important to note that the performance of machine learning algorithms highly depends on the relevant information contained in the data. Indeed, entropy is the core concept in Information Theory that provides a measure of the information content or the information gain widely used in Machine Learning and Data Science [29].

Machine Learning algorithms are being used in different applications to make important decisions that influence our daily lives: from health insurances contracts and their coverages, research spending prioritization, college admissions, to the way people find jobs or get loan applications approved. However, the models being used today sometimes do not take into account context and they have gender bias reinforcing discrimination conditions [30].

Given this situation, institutions such as the European Commission are working on gendered innovation, which aims at including the gender/sex analysis and gender diversity among research and innovation and is one of the Responsible Research and Innovation (RRI) indicators in the field of Science and Technology considered by the European Union. In fact, ensuring the incorporation of the gender perspective supposes a crosscutting issue for the European Commission in its Horizon 2020 funding program in every stage of the research process, where not only diversity in research teams needs to be fulfilled but also diversity in research methods and in research questions [31]. 

Taking this into consideration, in the last years some projects have emerged such as the European COST action “GenderSTE” (The international European Cooperation in Science and Technology (COST) network GenderSTE (Gender, Science, Technology and Environment) http://www.cost.eu/about_cost/strategy/targeted_networks/genderste) [32] that supports networking and the dissemination of knowledge with a gender perspective, the “GEECCO” (Gender Equality in Engineering through Communication and Commitment https://www.genderste.eu/) H2020 project focused on Engineering, or the initiative “gendered innovations” [28] led by Professor Londa Schiebinger and funded by the US National Science Foundation and the European Commission. Schiebinger states that gendered innovation perspective can benefit excellence in research, policy, and practice in many fields, from Health and Medicine or Social Sciences to Engineering and Technology [18]. 

In particular, Machine Learning and Data Science areas suppose new opportunities to include gendered innovation in Information Theory because they can be applied to many different domains, and there is an increase in demand for new data scientists. 

Data Science jobs have an important consulting component, beginning with a business context understanding phase to define goals and create a project plan. As it has been reviewed in the introduction, the participation of women in work teams as consultants, designers, or producers of technology is key for achieving high levels of creativity and collective intelligence, helping to avoid biases in research related to a lack of diversity and gender perspective. 

Some effects of not taking into account gender and sex are described in References [18,28,33,34,35,36,37,38,39,40], in the context of Machine Translation and Natural Language Processing algorithms, software development, smart cities, or robotics.

## 3. Decreasing the Gender Diversity Gap: Working Program

**Figure 1:**
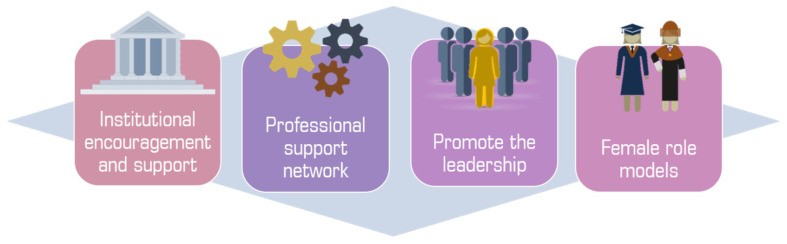
ETSE-UV main actions targeting the gender diversity problem.

In this Section we present the ETSE-UV working program, which is organized around four main actions, illustrated in Figure 1: (1) to provide institutional encouragement and support, (2) to increase the professional support network, (3) to promote and support the leadership and (4) to increase the visibility of female role models. 

### 3.1. ETSE-UV: Academic Environment and Support for the Program 

The ETSE-UV concentrates the engineering studies of the University of Valencia, namely, Chemical Engineering, Computer Science Engineering, Industrial Electronic Engineering, Multimedia Engineering, Telecommunications Electronic Engineering, Telematics Engineering, and Data Science. In this sense, the ETSE-UV faces a considerable challenge when dealing with the reduction of the gender gap in STEM both at the student and academic and professional levels due to the broad-spectrum of the offered degrees. Specialized faculties or schools focusing on a given degree, on the other hand, can also apply for this program with a considerably smaller effort.

The ETSE-UV, as a higher education institution, can contribute to the gender gap reduction in STEM in two ways: First, interacting with secondary schools with the aim of fighting stereotypes—with female role models—and highlighting the applicability of the different STEM branches, and secondly, retaining and engaging female students once they have accessed the studies. This second action needs to conjugate the use of female role models and activities helping the students to build strong support networks during their studies. In a third axis, the institution should try to help as much as possible its graduates to find support networks across their professional careers. 

The program of the ETSE-UV targets some of the barriers stated in Reference [1], and it is aligned with the interventions proposed by UNESCO in Reference [8], specifically the extra-curricular student engagement, the mentorship, and role models. The four main actions of the program illustrated in Figure 1 are supported by conclusions or evidence arising from the long literature on the topic, such as in References [19,20,21,23,41,42,43,44,45,46]. As UNESCO points out in Reference [8], promoting more female role models in STEM fields, more precisely, female students and faculty members in higher education, is an important strategy to attract women and girls into STEM fields. Report [8] also highlights the need for support programs and initiatives for female STEM professionals. 

Please note that the program presented in this paper is an exploratory initiative that started in 2011, while countries such as the United States have already implemented STEM intervention programs on several college campuses during the last years. These programs are designed to increase the participation of under-represented students in STEM fields, which includes women. We refer the reader to Reference [41] for a description of several programs currently working at different colleges, focusing on strategies such as academic advising, faculty mentorship, tutoring, internship opportunities, and career and skill development. The program evaluated in Reference [41] hypothesized that perceived benefits of STEM intervention programs should be related to an engineering major confidence, which at the end relates to the persistence in the STEM field. Basically, they focused on evaluating the impact of the program on the self-confidence or self-efficacy of the female students, which has been proved to be a fundamental indicator of STEM performance and perseverance [42,43]. Reference [42] found out that even if some programs were not designed to increase STEM self-efficacy, there is was a direct correlation between increased performance and an increase in self-efficacy. In Reference [42], some interventions are recommended to increase the self-efficacy at different educational levels and contexts, namely, the use of mastery experience, vicarious experience, social persuasion, and the consideration of physiological reactions. Finally, Reference [44] also provides evidence-based recommendations for best practices to improve STEM diversity, and, interestingly, highlights that there is still a need to understand how to translate research in this field into evidence-based interventions. As a future research direction, the authors of Reference [44] recommend targeting not only women’s attitudes toward STEM but also family, and STEM faculty and employers.

To the best of our knowledge, this program is a unique initiative in Spain in the sense that (i) it started with the beginning of Bologna compliant bachelor degrees (2011) and (ii) it goes beyond the formal requirement of an equality plan definition, encompassing the three dimensions where the ETSE-UV (or a higher education institution) can apply some influence: Students in the years prior to the University, students already enrolled in STEM disciplines, and new graduates in STEM branches. The problem of equality plans at the global institution level (the University of Valencia, in our case) is that they give general recommendations, whereas reports such as in Reference [8] are not specifically addressing the higher education ecosystem (as we have previously identified, the primary target group is located in the range of 6 to 15 years old).

### 3.2. Actions and Recommendations for Practice 

In this subsection, we describe a set of activities per action which are straightforward to implement with basic budget allocation, and which allow motivating and retaining female students, as well as integrating their male colleagues. In the ETSE-UV case, a budget of 1500 euros are allocated each year for the promotion of student activities. In addition to this primary funding, faculty members and student associations apply for external funding, e.g., from the University Equality Unit or the central Student Services. This means that there is a certain variation in the available funding over the years. 

This program relies heavily on the commitment of the faculty members, especially from the school’s board, which is both an advantage and a drawback, since a change in the board may imply more efforts from the faculty members in order to carry out this program and the different actions. Note also that for a faculty member, this type of ‘extra-curricular’ work is not reporting any benefits in terms of future promotion, for example. Hence, participating in these activities requires time from the faculty members, as well as facing sometimes the opposition of Department colleagues. 

In general, a set of recommendations is followed when organizing an activity for the program:

- Interaction between teachers and students is encouraged
- Participation of both female teachers and students is sought (targeting at least the 60 (male)/40 (female) ratio)
- Scheduling of at least two activities per year
- Broadcasting of the activities through the institutional webpage as well as the social networks
- Integration of male students is mandatory

This last recommendation is fundamental to avoid frustrated feelings among the male students, as it has been discussed in the introduction. One specific target of the programmed activities is the increase of self-efficacy or self-confidence, since women in engineering is a group with lower levels of self-efficacy [41,42,43], and as stated before, an increased self-efficacy leads to an enhanced persistence in the STEM field. Finally, in order to keep the momentum, at least two activities need to be scheduled during the academic year, following the hypothesis that to position an academic institution in a leading position, and to actually achieve a long-term change in the diversity gap, a continuous and sustained effort needs to be done in this direction, creating an academic environment more inclusive, welcoming, and supporting [19,20,21]. 

In the following, we describe the activities carried out by the program. Links to some of the actions are provided in Appendix A.

A. Providing Institutional Encouragement and Support

The objective of this action is to increase the number of female students, providing also support and engaging them, with the aim of preventing them from resigning in the early stages. The main activities under this objective can be divided as follows:

- *Promoting interaction with high school students through workshops and seminars*. In this case, high school students are the targeted group, and the location of the activity is a key factor: (i) The workshops take place in the ETSE-UV. Training options and professional opportunities in the fields of Information Technology, Multimedia and Telecommunications are presented and short laboratory sessions are also scheduled; (ii) the seminars are given at high schools, where female faculty members perform talks about topics related to the ETSE-UV expertise.

Note that the presence of female faculty members is fundamental to establish female role models. On the one hand, there are many studies supporting the evidence that female teachers have a beneficial effect on girls’ perceptions, interest, and self-confidence in STEM subjects and STEM career aims (see for example References [8,45,46]). On the other hand, some references could not find a direct correlation between female teachers and girls’ STEM performance, pointing out other factors [47,48]. However, even these last studies found that female teachers have a positive influence both on girls and boys. The fact that female faculty members visit high schools, in addition to establishing role models, can be considered a social persuasion intervention [42], since it is an opportunity to emphasize that STEM fields are opened to both female and male students.

- *Promoting interaction between female faculty members and University students.* In this second category, female students already enrolled in STEM studies are the targeted group. Actions in this category are aligned with results stated in [8], which show that interactions between teachers and students influence girls’ engagement, self-confidence, performance, and persistence in STEM studies, as well as with Reference [43], which highlights student/professor relationships as fundamental in order to retain engineering students. Several activities can be carried out in the framework of international events (International Day of Women and Girls in Science or the Ada Lovelace Day, for example), such as building a photograph panel with women studying and working in the institution or broadcasting on social networks joint pictures of female students, researchers, and faculty members. In this category, the main activity is the *+Dones* action, a YouTube contest where female students or staff share why they decided to join the institution and how they would increase the number of women in ICT or STEM studies.

B. Increasing the Professional Support Network

The objective of this action is to increase the presence of women in all stages of STEM professions. As it has been stated in the introduction, one of the barriers facing women is the lack of a strong support network in the workplace, experiencing even gender bias at some point. This action is aligned with research initiatives such as the Leadership Lab [49], designed with the focus of increasing and retaining women in STEM professions. The main activities under this objective are the following:

- *Promoting interaction with professional women working in STEM environments.* Workshops or panels with women from different fields of engineering can be held, encouraging the presence of women in studies and professional engineering activities. Examples of successful or rewarding work-life balances are seminal to empower female students. Participation of international professional networks in the panels, such as the Institute of Electrical and Electronic Engineers (IEEE), should be encouraged. Following the definition from Reference [42], this activity can be classified as a vicarious experience intervention for self-efficacy improvement. In this category, we would like to highlight the initiative *DataBeersVLC* (belonging to the global not-for-profit initiative DataBeers), which propitiates encounters among data scientists and data users from different entities (industry, government, academia, etc.). The organizers of the initiative, related to the ETSE-UV, noted that it was extremely difficult to find female speakers and special sessions with exclusively female speakers are being scheduled.
- *Building female student support networks.* Research shows that women’s STEM organizations provide female students with a positive sense of community, improved self-efficacy [41], and an increased engineering identity, among others [21]. Women not involved in these support systems tend to form their own informal support network, but they do not have access to exclusive resources available to these organizations. The authors in Reference [21] established that there is a gap between structural and cultural acceptance, meaning that, even if women are aware that STEM organizations are a useful support resource, they refrain from joining to avoid seeming less-sufficient than their male colleagues. In the ETSE-UV case, the IEEE Women in Engineering (WIE) Student Branch Affinity Group started in 2018, with the mission of encouraging girls into STEM careers and creating support networks for women already working in STEM.

C. Promoting and Supporting the Leadership

The objective of this action is to increase the presence of women in high-rank academic positions, addressing what it is known as vertical segregation. Note that research in this field suggests that, in order to achieve a more gender-balanced academic environment, opportunities for women’s leadership need to be created [21]. In the ETSE-UV case, the participation of women on the governing boards has been encouraged. In 2010, 28.5% of the decision-making positions were led by women. In 2018, this percentage has reached the value of 40.6% (defined in the Spanish Equality Law as an equilibrium threshold), engaging and supporting female faculties in the development of leading roles. Following the Statutes of the University of Valencia, an equality committee has been established, dealing with all aspects related to gender equality and diversity.

D. Increasing the Visibility of Female Role Models

The objective of this action is to recognize and disseminate the achievements of women in STEM fields. The main activities attempting this objective are the establishment of recognitions to pioneering women in the institution and giving awards to female students with outstanding final degree projects.

## 4. ETSE-UV: Case Study

In this Section, quantitative and qualitative indicators supporting the ETSE-UV working plan are presented. The evaluation of a program of this type is a research field itself, and not many references can be found providing indicators based on objective metrics. For example, Reference [21] examines the gendered experiences of women graduated in engineering both in academia and in companies. They established a set of participants, from different fields, and realized a series of interviews which were used to search for patterns. In Reference [49], a research-based leadership development program is presented, designed with the aim of increasing and retaining women in STEM professions. This program targets women which are already in professional environments and its outcomes are evaluated based on the feedback from the participants. In Reference [41], evaluation of the hypothesis is done via cohort surveys at different stages of the program until student’s graduation. Report [50] analyzes the evolution of the number of registered female students in ICT-related engineering (mainly Computer Science and similar degrees), considering the case of Spain. The authors also face the problem of quantifying the impact of affirmative actions towards promoting the enrollment of female students. In their paper, they establish a set of metrics to quantify: Evolution and percentages of female students, possible discontinuity points, percentages from other universities and trends in the results. Note that this methodology was also used in Reference [51]. To evaluate the ETSE-UV case, metrics have been obtained following Reference [50], as well as registering qualitative outcomes directly related to the different actions performed over time. 

Starting with the Bachelor level, Table 2 compares the evolution of the percentage of enrolled female students in ICT-related degrees. The second column collects the aggregated data for the Pre-Bologna degrees at the ETSE-UV, namely, Computer Science Engineering, Electronic Engineering, Telecommunications Electronic Engineering and Telematics Engineering, while the fourth and fifth columns show the aggregated data for the Bologna compliant degrees at the Spanish and ETSE-UV levels, respectively. The program presented in this paper started in 2011, targeting the ETSE-UV’s Bologna compliant degrees (Computer Science Engineering, Multimedia Engineering, Industrial Electronic Engineering, Telecommunications Electronic Engineering, and Telematics Engineering). Therefore, Table 2 shows aggregated data and the pre and post program’s situation. Spanish data for the ICT category reflects that the percentage of enrolled female students decreases year after year. In general, ETSE-UV is positioned above the Spanish reference value both on average and each year. This is also supported in Reference [50], which states that the ETSE-UV is one of the Spanish Engineering Schools with a larger percentage of female students in the ICT field. Actually, it is the third University in Spain according to the percentage of women enrolled in ICT studies, and the second one in terms of the trend of this indicator.

**Figure 2:**
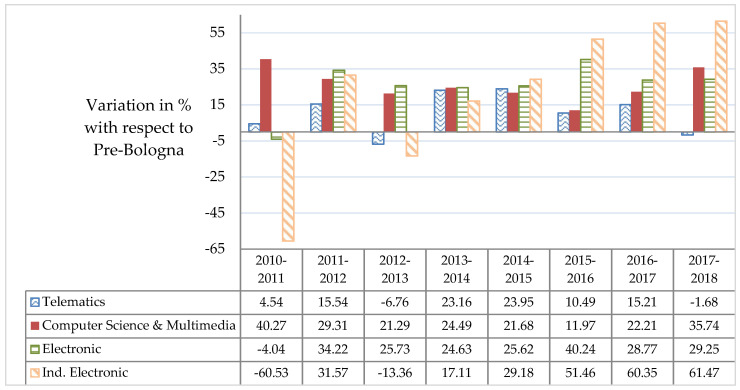
Comparison of the pre/post program situation. Variation in % of the percentage of enrolled female students with respect to the average percentage of female students enrolled in Pre-Bologna degrees for the period 2006–2010 (14.53% in Telematics Engineering, 13.88% in Computer Science Engineering, 10.97% in Telecommunications Electronic Engineering and 5.63% in Electronic Engineering).

Disaggregating the data into the different degrees, Figure 2 shows the variation (%) of the percentage of enrolled female students with respect to the average percentage of female students enrolled in Pre-Bologna degrees (period 2006–2010). The reference average percentage values for the period 2006–2010 stand in a 14.53% in Telematics Engineering, 13.88% in Computer Science Engineering, 10.97% in Telecommunications Electronic Engineering, and 5.63% in Electronic Engineering, according to Reference [22]. Data of Computer Science Engineering and Multimedia Engineering have been aggregated in the 2010–2018 period to establish a fair comparison, following the categories established in Reference [52].

To understand results from Figure 2, one needs to bear in mind that the initial window 2010–2011/2012–2013 can be regarded as a transient period in the implantation of the Bologna compliant degrees. Regarding the pre/post situation, Figure 2 proves that, in general, the percentage of female students is above the reference value (Pre-Bologna) during the evaluated period. Electronic, Computer Science and Multimedia Engineering show steady or positive trends, while the oscillations presented by Telematics Engineering indicate that a larger effort is needed in this degree. In any case, this exploratory analysis would benefit from the availability of a larger data window.

We have also performed a chi-square (χ^2^) test of independence in order to evaluate whether there is a significant association between the categories of the variables *Sex* (female, male) and *Program* (pre, post) at a 0.05 significance level, which is summarized in Table 3. Results indicate that the relation between these variables was significant when considering the aggregated values of all ETSE-UV’s degrees ($\chi^{2}\left(1\right)=10.274$, *p* < 0.01), as well as for Computer Science and Multimedia Engineering ($\chi^{2}\left(1\right)=10.929$, *p* < 0.001), Telematics Engineering ($\chi^{2}\left(1\right)=5.3308$, *p* < 0.05) and Electronic Engineering ($\chi^{2}\left(1\right)=6.7095$, *p* < 0.01). Data show evidence that the percentages of female students vary between the different levels of the variable *Program*, with the exception of Industrial Electronic Engineering, which was not significant despite the increments shown in Figure 2 and Table 3. Evidence proves that these degrees are male-dominated, although a positive and significant effect is observed in the post program time period. 

**Figure 3:**
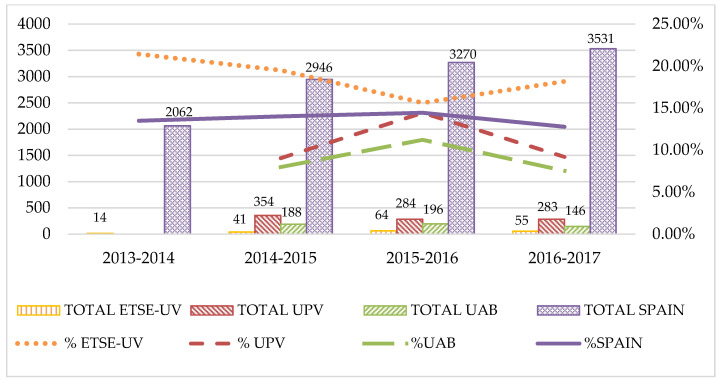
(Left axis) Total number of students graduating in Computer Science and related engineering degrees (male and female) are represented with bars for ETSE-UV, Universitat Politècnica de València (UPV), Universitat Autònoma de Barcelona (UAB), and Spain cases, respectively. (Right axis) Percentages of female graduated students in the four cases are represented with lines.

Regarding the percentage of graduated female students, statistics obtained from Reference [22] show that aggregating the values for all the Bologna compliant degrees and focusing on the 2013–2014/2017–2018 window, the ETSE-UV achieves an average value of 15.12% graduated female students. Note that this time period corresponds to the years when the first Bologna graduates were obtained, since Bologna compliant degrees establish a four year’s duration. To benchmark this value, data has been collected from universities with similar characteristics (both from the Mediterranean region) [52]. The first one is the Universitat Politècnica de València (UPV), a technical University also located in the city of Valencia. However, in this case, each faculty or school is specialized in a given degree. The second one is the Universitat Autònoma de Barcelona (UAB), located in Barcelona. This University also focuses the engineering studies in a technical school, facing similar challenges to the ETSE-UV as it has to compete for students with other universities placed in Barcelona. Note that data from UPV and UAB were not disaggregated by sex until 2014, and that data for 2017–2018 are not available yet [52], thus the actual comparison window spans from 2014–2015 till 2016–2017. Figure 3 shows, on the one hand, the evolution of the percentage of graduated female students for the ETSE-UV, UPV and UAB cases, as well as the Spanish value as a baseline. On the other hand, Figure 3 also includes the total number of graduated students (male and female) in the four cases, showing that the ETSE-UV, apart from exceeding UPV, UAB and the Spanish reference values, is at least maintaining the percentage of graduated female students. In order to establish a fair comparison, only data corresponding to Computer Science and Multimedia Engineering is considered in this evaluation.

ETSE-UV’s program is also addressing Master students from Bioinformatic, Telecommunications Engineering and Services, and Web Applications Engineering, as well as PhD students from the PhD program of information technologies, communications, and computation. However, we had difficulties finding a common benchmark to display the data due to the disparity in the offered studies and the coexistence of compliant and non-compliant Bologna degrees in the period under analysis. On average, for the 2010–2018 period, the aggregation of Master and PhD data indicates that a 35.36% of the graduated students were female. This value can be framed in the UVEG global context, where the average percentage of female students graduating in Master and PhD levels was a 38.82% in 2016–2017.

Besides the quantitative results, two qualitative outcomes of the ETSE-UV program can be highlighted: The reception of the “premio equit@T” in 2018, an award from the Universitat Oberta de Catalunya recognizing the effort in promoting the presence of women in academia as well as in ICT companies, and the inspiration and engagement provided by the *+Dones* activity, which has been reflected by the publication of one article authored by one of the students entering the contest [53].

As part of our future research, longer series of data will be collected in the upcoming years to confirm the trends observed in the above analysis. In addition, subjective indicators based on experiences such as those reported in References [21] or [41] will be integrated, for example by tracking a target group of students during their academic career, performing regular interviews, and tailored surveys each academic year. In this way, the impact of the program in terms of self-efficacy improvement can be properly evaluated, as well as trying to establish correlation or inferences from the collected data.

## 5. Conclusions

The gender diversity gap in science, technology, engineering and mathematics (STEM) fields has been presented in this paper as a problem composed of many factors. Each one of these factors has a different impact and would require the action of several actors and institutions in order to achieve a real change towards a gender sensitive culture. The School of Engineering from the University of Valencia, Spain, has been focusing on four main actions, which have allowed achieving a percentage of graduated female students from information and communications technology studies above the Spanish reference values, as well as reaching 40% of top-decision positions held by faculty women. One action driven to reduce the gender gap is the gendered innovation initiative. While not all research must necessarily have a gender perspective, we believe that some areas of the Information Theory field can benefit from it. To conclude, in order to improve the proportion of women working in the Information Theory field, additional effort must be done in explaining its impact outside of the research community, as well as increasing the visibility of women already working in the field. 

## Figures and Tables

**Table 1:** Percentage of women according to their academic position in the University of Valencia (UVEG) and in the School of Engineering at the University of Valencia (ETSE-UV) (2017–2018) [22].

Position	UVEG	ETSE-UV
Full Professor	28.12%	16.67%
Associate Professor	44.85%	21.51%
Assistant Professor	46.96%	21.21%
Total	43.28%	20.90%

**Table 2:** Comparison of the pre/post percentage (%) of enrolled female students in the ETSE-UV. The second column shows the evolution in Pre-Bologna studies [22], while the fourth and fifth columns show the evolution in Spain [52] and at the ETSE-UV [22], respectively, for Bologna compliant degrees.

Year	ETSE-UV ^1^	Year	ICT (Spain)	ETSE-UV ^2^
2006–2007	12.96	2010–2011	13.36	13.55
2007–2008	12.53	2011–2012	13.33	15.28
2008–2009	12.57	2012–2013	13.04	13.49
2009–2010	12.31	2013–2014	12.89	14.69
		2014–2015	12.6	14.52
		2015–2016	12.00	14.12
		2016–2017	12.06	14.56
		2017–2018	Not available	15.02
**Average**	**12.59**	**Average**	**12.75**	**14.41**

^1^ Aggregated data for Computer Science Engineering, Electronic Engineering, Telecommunications Electronic Engineering, and Telematics Engineering. ^2^ Aggregated data for Computer Science Engineering, Multimedia Engineering, Industrial Electronic Engineering, Telecommunications Electronic Engineering, and Telematics Engineering.

**Table 3:** Relation between sex and program intervention. Raw numbers as well as % of female and male students are included in the pre and post columns.

Degrees	Pre (%)	Program Post (%)	*p*-Value ^a^
**All Degrees**			**<0.05**
Female	712 (**12.6%**)	1126 (**14.5%**)	
Male	4938 (87.4%)	6623 (85.5%)	
**Comp. Sci. & Mult.**			**<0.001**
Female	293 (**13.9%**)	520 (**17.3%**)	
Male	1814 (86.1%)	6623 (82.7%)	
**Electronic**			**<0.01**
Female	208 (**11.0%**)	247 (**13.8%**)	
Male	1687 (89%)	1545 (86.2%)	
**Ind. Electronic**			0.1582
Female	18 (**5.6%**)	118 (**7.9%**)	
Male	301 (94.4%)	1368 (92.1%)	
**Telematics**			**<0.05**
Female	193 (**14.5%**)	245 (**17.8%**)	
Male	1136 (85.5%)	1132 (82.2%)	

^a^*p*-value calculated by the chi-square test of independence, where text in bold indicates a statistically significant difference with a *p*-value less than 0.05.

## Appendix A

Examples of secondary workshops: http://ir.uv.es/ceP2Wrx and http://ir.uv.es/pwfjt0J. Activities related to the International Day of Women and Girls in Science: http://ir.uv.es/XmXl7nD. YouTube videos submitted to *+Dones* action are available online (Spanish version) at http://go.uv.es/actosetse/vota+Dones. Women and Engineering Workshop: http://ir.uv.es/vFS4yDp. DataBeersVLC: http://ir.uv.es/ORw5tbt. Ada Lovelace Day: http://ir.uv.es/M3DXjig and http://ir.uv.es/M3DXjig. IEEE WIE: http://ir.uv.es/bzCa6zE. Equality Commitee: http://ir.uv.es/VhM3tBO. Institutional recognition: http://ir.uv.es/ryy9NMF. Award—Premio equit@T: http://premi-equitat.uoc.edu/es/. Note that part of these materials was published in Spanish.
